# Three-dimensional super-resolution protein localization correlated with vitrified cellular context

**DOI:** 10.1038/srep13017

**Published:** 2015-10-14

**Authors:** Bei Liu, Yanhong Xue, Wei Zhao, Yan Chen, Chunyan Fan, Lusheng Gu, Yongdeng Zhang, Xiang Zhang, Lei Sun, Xiaojun Huang, Wei Ding, Fei Sun, Wei Ji, Tao Xu

**Affiliations:** 1 National Laboratory of Biomacromolecules, Institute of Biophysics, Chinese Academy of Sciences, Beijing, 100101, China; 2 College of Life Science and Technology, Huazhong University of Science and Technology, Wuhan, Hubei, 430074, China; 3 College of Life Sciences, University of the Chinese Academy of Sciences, Beijing, 100049, China; 4 Center for Biological Imaging, Institute of Biophysics, Chinese Academy of Sciences, Beijing, 100101, China

## Abstract

We demonstrate the use of cryogenic super-resolution correlative light and electron microscopy (csCLEM) to precisely determine the spatial relationship between proteins and their native cellular structures. Several fluorescent proteins (FPs) were found to be photoswitchable and emitted far more photons under our cryogenic imaging condition, resulting in higher localization precision which is comparable to ambient super-resolution imaging. Vitrified specimens were prepared by high pressure freezing and cryo-sectioning to maintain a near-native state with better fluorescence preservation. A 2-3-fold improvement of resolution over the recent reports was achieved due to the photon budget performance of screening out Dronpa and optimized imaging conditions, even with thin sections which is at a disadvantage when calculate the structure resolution from label density. We extended csCLEM to mammalian cells by introducing cryo-sectioning and observed good correlation of a mitochondrial protein with the mitochondrial outer membrane at nanometer resolution in three dimensions.

A major goal in cell biology is to locate and directly visualize macromolecules in their native cellular context, i.e., visual proteomics^1^. Although immuno-electron microscopy (EM) can be employed to label proteins, it suffers from low label density and poor preservation of cellular morphology. Recently developed super-resolution fluorescence microscopy (i.e., STED, (f)PALM/STORM, SIM) techniques have achieved nanometer resolution of fluorescence-labeled proteins^2^. However, super-resolution fluorescence microscopy cannot reveal the ultra-structural context. The integration of super-resolution fluorescence microscopy with EM may allow the correlation of fluorescently labeled individual proteins with the cellular ultrastructure at nanometer precision and holds great promise for meeting the technique demands of biological researches.

Indeed, STED and (f)PALM have been combined with EM to correlate protein localization and ultrastructural features^3^,^4^,^5^. Unfortunately, the chemical fixation and staining procedures during EM sample preparation tend to distort native cellular structures^6^,^7^ and quench fluorophores^8^,^9^. High pressure freezing followed by freeze substitution has been proved to maintain fluorescence, and meanwhile largely keep the cellular structural details^5^. However, this procedure needs to balance the contrast of EM imaging and fluorescence preserving during the staining process. Normally, post staining after fluorescence imaging is required to enhance the contrast in EM, which may cause further distortion of the sample and affect the precision of correlation^5^,^9^. Recently, an improvement of the sample preparation procedure of high pressure freezing, freeze substitution and resin embedding was described to better preserve fluorescence and photo-switching of standard fluorescent proteins, such as mGFP, mVenus and mRuby2^10^. This improved procedure enabled the correlation of fluorescently labeled structures to the ultrastructure in the same cell at higher precision and superior structural preservation. An alternative sample preparation method is vitrification, which preserves the structures in a near-native state in glasslike amorphous ice with no compromise of fluorescence preserving^11^,^12^. Another advantage of vitrification is the decreased photobleaching at cryo-temperatures^13^,^14^,^15^. Hence, there is now an emerging demand for fluorescence microscopy under cryo-conditions (cryo-FM) and its integration with other techniques including cryo-EM^15^,^16^,^17^.

The limited sample thickness of cryo-EM and the low resolution of cryo-FM hamper the application of CLEM with vitrified samples. Cryo-electron microscopy of vitreous sections (CEMOVIS) is the most promising technique to visualize the three-dimensional (3D) architecture of hydrated cells and tissues which is too thick for cryo-EM^18^,^19^. Cryo-FM have been used to guide EM data acquisition with vitreous sections^20^,^21^. Several technical challenges limit the resolution of cryo-FM, such as low NA objectives, poor mechanical stability of the cryo-stage, and undesired performance of fluorophores under cryo-conditions. The adaptation of super-resolution methods to cryo-conditions is expected to overcome the resolution limit of cryo-FM. Indeed, two recent studies along this direction have extended cryo-FM to super-resolution imaging with a 3-5-fold improvement in lateral resolution^22^,^23^. Here, we further improve the resolution of cryo-FM to be comparable with ambient super-resolution imaging and extend csCLEM to 3D for mammalian cells by employing cryo-sectioning.

## Results

### Cryo-nanoscopy setup

**Figure 1:**
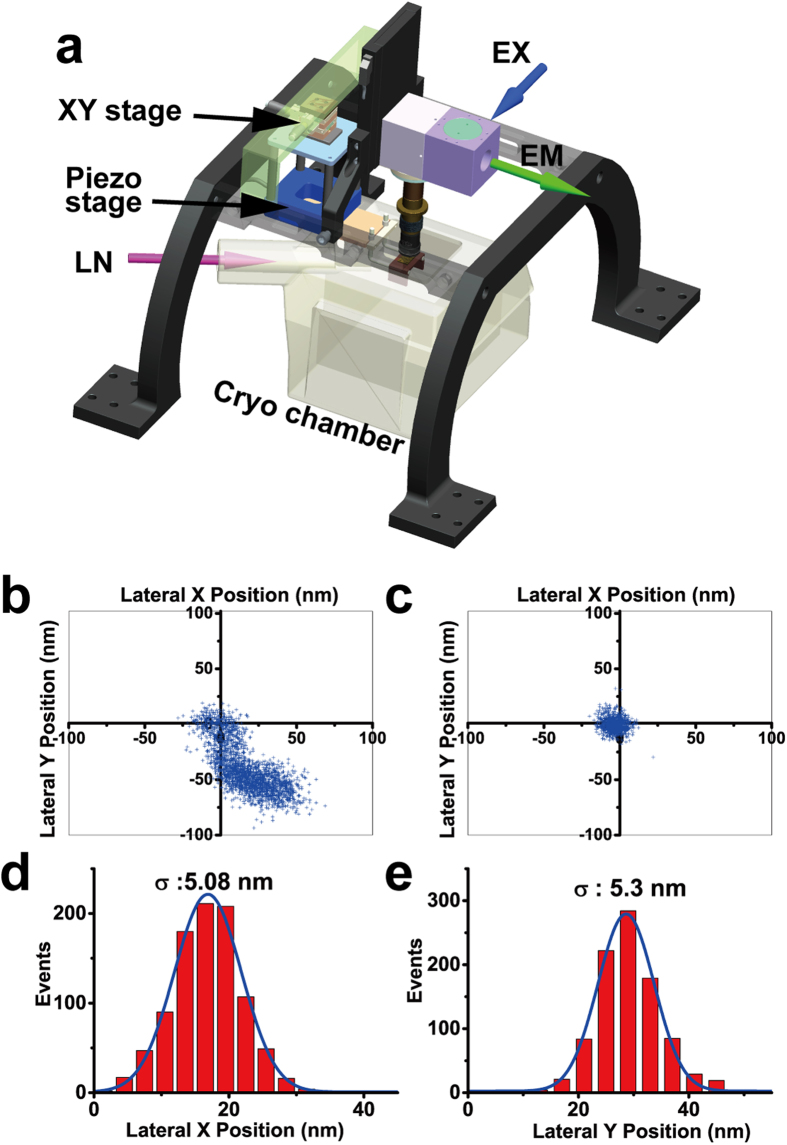
Schematic drawing and drift correction capability of the system. (**a**) Schematic drawing of the cryo-nanoscopy system. A dark red fluorescent bead was monitored for 5 min without active feedback (**b**) and with active feedback (**c**). Lateral X (**d**) and Y (**e**) position distribution of all points in (**c**). Data show that the active feedback stabilization system could effectively suppress the drift. Figure 1a was drawn by Wei Ji.

Our goal is to correlate genetically labeled proteins with subcellular structures of mammalian cells at nanometer precision. Mammalian cells are generally too thick for EM. Therefore, we cryo-sectioned mammalian cells to slices of ~200 nm thickness and employed CEMOVIS, which enables us to observe the internal architecture of cells or even subcellular organelles in their fully hydrated state^18^,^19^. We employed the strategy of single-molecule localization microscopy (SMLM, i.e., (f)PALM/STORM) for super-resolution imaging under cryo-conditions. The long imaging time required for SMLM necessitates long-term mechanical and thermal stability of the cryo-stage to hold the cryo-sections. To meet the above requirements, we built a cryo-nanoscopy system, as depicted in Fig. 1a. We used a special designed upright microscope coupled with a commercialized cryo-chamber (EM FC6, Leica) (Fig. 1a and **Methods**, Figures S1, S2). The upper part of the objective was wrapped with heating wires to keep the lens warm and free of frost. The sample holder was fixed on a copper block by magnetic buttons. The cryo-chamber includes a self-pumping filled Dewar connected to the liquid nitrogen inlet and feedback systems to stabilize the temperature and nitrogen gas airflow. The sample holder was separated from the Dewar and immerged in cooled nitrogen gas, which greatly reduced vibrations caused by the flow and boiling of liquid nitrogen. We further introduced an active drift correction system to stabilize the position of the sample with nanometer precision (~5 nm) during long-term data acquisition as described in Methods section (Fig. 1b–e).

### Photophysical properties of FPs under cryogenic temperature

**Figure 2:**
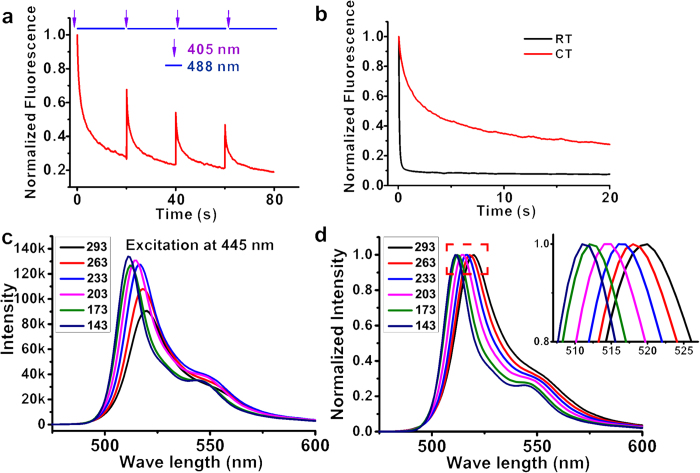
Photophysical properties of Dronpa at CT. (**a**) Photoswitching capability of Dronpa at CT (113 K). Purified Dronpa proteins were first switched off by a 488 nm laser, followed by alternative illumination with 405 nm and 488 nm lasers. Each purple arrow indicates a 5 s 405 nm laser pulse. (**b**) Fluorescence decay kinetics of Dronpa at RT and CT under the irradiation of a 1.5 kW·cm^−2^ 488 nm laser. The decay curves at RT and CT were both fitted with a bi-exponential function^27^,^28^. The time constants/amplitudes at RT are 0.06 s/0.87 (fast phase) and 2.37 s/0.04 (slow phase), and the corresponding values at CT are 0.87 s/0.37 (fast phase) and 8.32 s/0.33 (slow phase). Please note that at RT, the amplitude of the slow phase is very small (4%), indicating fast bleaching and off-switching mainly in the fast phase (**c**,**d**). (**c**) shows the evolution of the emission spectra of Dronpa subjected to a temperature gradient from 293 K to 143 K, (**d**) is the normalized presentation, and the inset is the magnified view of the red area in (**d**).

Another key requirement of cryo-SMLM is the ability of fluorescent probes to be photoswitched. To find suitable photoswitchable FPs that function at cryogenic temperature (CT), we first investigated the temperature-dependent behavior of FPs including Dronpa^24^, mGeos-M, mEos3.2, PATagRFP and PAmCherry. We found that mEos3.2, an irreversible photoconvertor with the best performance at room temperature (RT)^25^, required >100-fold more power to be efficiently photoconverted from green to red by a 405 nm laser at CT (**Methods**, Figure S3). For the reversibly switchable fluorescent proteins, such as Dronpa, reversible photoswitching was maintained at CT (Fig. 2a). We also compared the ensemble photobleaching of Dronpa at RT and CT under the same laser power. Dronpa showed much greater anti-bleaching ability at CT than at RT (Fig. 2b). The temperature dependence of the fluorescence emission spectra of Dronpa was measured to guide the selection of suitable filter sets (see **Methods** section for experimental detail). The emission spectrum of Dronpa displayed a blue shift and narrowing band with decreasing temperature, a behavior also observed for GFP proteins^26^ (Fig. 2c,d). EGFP also displayed reversible photoswitching behavior at CT, as previously reported for various GFP mutants^23^,^27^,^28^ (**Methods**, Figure S4).

We screened the photon budget performance of these photoswitchable FPs which are commonly used under our CT imaging conditions with purified fluorescent proteins (see **Methods** section). The setting temperature of cryo-chamber is 113 K which was verified with a tiny Pt100 temperature sensor placed around the sample. The real temperature may be slightly higher than the setting point due to laser illumination. We found that, among these candidates, Dronpa exhibited the best performance in terms of photon budget and localization precision for single-molecule imaging (Table 1). Hence, we selected Dronpa for the following experiments.

### Verifying resolving capability of cryo-nanoscopy system

We further tested the performance of Dronpa in an intracellular context at CT and verified the resolving capability of our cryo-nanoscopy system. We constructed a marker for the mitochondrial outer membrane by fusing Dronpa with the first 33 amino acids of TOM20 (see **Methods** section), which is responsible for localizing TOM20 to the mitochondria^5^. The proper localization of TOM20-Dronpa in HEK293 cells was verified using confocal microscopy (**Methods**, Figure S5).

**Figure 3:**
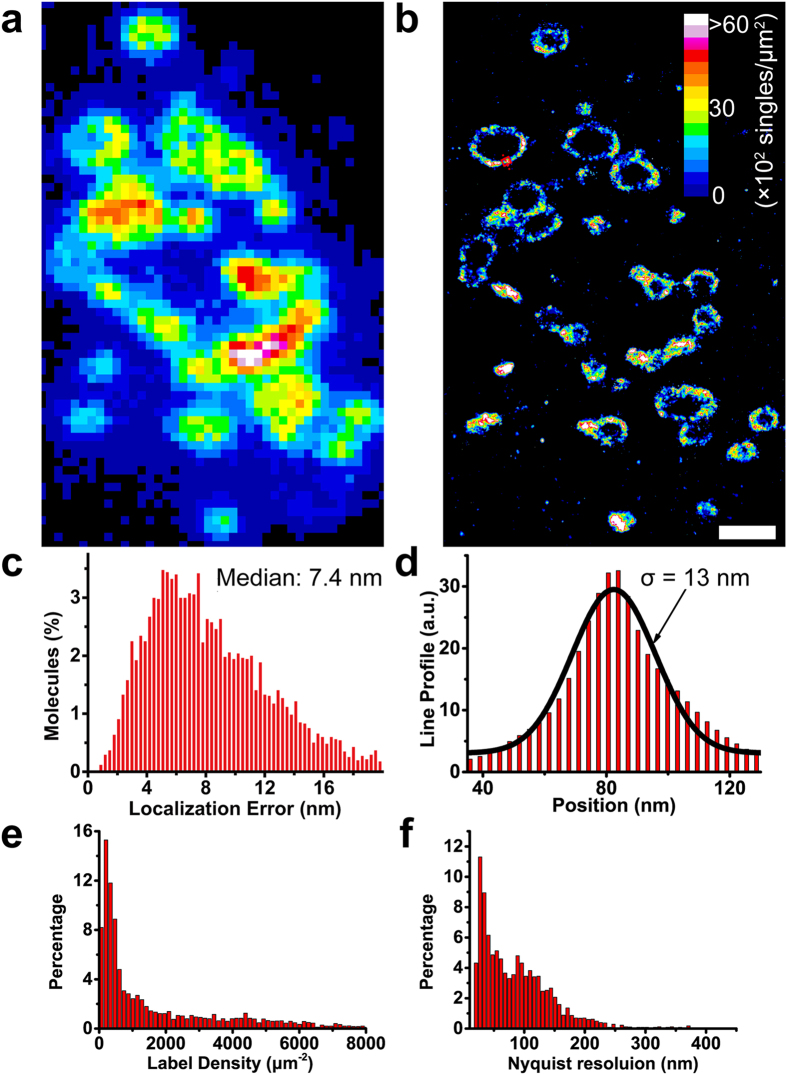
Verification of cryo-nanoscopy’s resolving capability. (**a**,**b**) Conventional fluorescence image (**a**) and PALM image (**b**) of a 200 nm cryo-section of a TOM20-Dronpa-labeled HEK293 cell. Scale bar, 1 μm. (**c**) Distribution of the localization error in the middle layer of z-sections obtained from 3D data in (**b**). (**d**) Line profile of the position marked in (**b**) (red). The middle layer was used to generate the line profile. (**e**) Distribution of label density in (**b**), 1850/744 μm^−2^ (mean/median). (**f**) Distribution of Nyquist resolution calculated from the label density in (**e**), 46/74 nm (mean/median).

After high-pressure freezing and cryo-sectioning (experimental procedurals described in **Methods** section), we collected ~200 nm thick cryo-sections of HEK293 cells expressing TOM20-Dronpa (Fig. 3a) on TEM finder grids and imaged them using our cryo-nanoscopy. The challenge is to maintain the vitrified state of the specimens during super-resolution fluorescence imaging. The fluorescence collection efficiency of our cryo-nanoscopy was lower than standard super-resolution microscopy at RT due to the use of an air objective (NA 0.8), so we increased the excitation laser power to achieve sufficiently bright single-molecule signals. However, higher laser power tends to heat the cryo-sections and devitrify the sample, as shown by the appearance of ice crystals. To reduce the laser absorption-induced heat, we replaced the high absorbance carbon film with a low background fluorescence formvar film (see **Methods** section). Due to low absorbance of the formvar film, we could increase the power of the 488 nm laser up to 1.5 kW·cm^−2^ at the specimen without devitrifying the samples (**Methods**, Figure S6). The reconstructed super-resolution images from ~20,000 frames of single-molecule data clearly revealed ring shapes representative of the outer membrane of mitochondria (Fig. 3b). To obtain 3D single-molecule localization information, we introduced a cylindrical lens as previously reported^29^ (**Methods**, Figure S7). The single molecules were distributed evenly along the z-axis with a full width at half maximum (FWHM) of 240 nm, corresponding nicely to the thickness (~200 nm) of the cryo-sections plus the uncertainty caused by limited photon numbers. We divided the total single-molecule data into 8 z-sections of ~30 nm thickness. For a single z-section in the middle, we estimated an average localization precision *s* of ~7.4 nm in the lateral direction (Fig. 3c). The z-axis resolution was estimated to be ~40 nm by analyzing the z distribution of long-lasting single molecules^29^. Further resolution improvement can be achieved by using better-localized fluorophores and applying an acceptable precision cutoff, as recently demonstrated^30^. The apparent width of a line profile of TOM20-Dronpa along the mitochondrial outer membrane could reach σ ~13 nm for a given z-section (Fig. 3d), which represents the summed consequence of limited photon count, residual mechanical drift, and labeling uncertainty of the TOM20-Dronpa protein. The label density was estimated to be 1850/744 μm^−2^ (mean/median) (Fig. 3e), leading to a Nyquist resolution of ~46/74 nm (Fig. 3f), which is a 2-3-fold improvement over the recent reports^22^,^23^.

### 3D Protein localization correlated with vitrified cellular context

**Figure 4:**
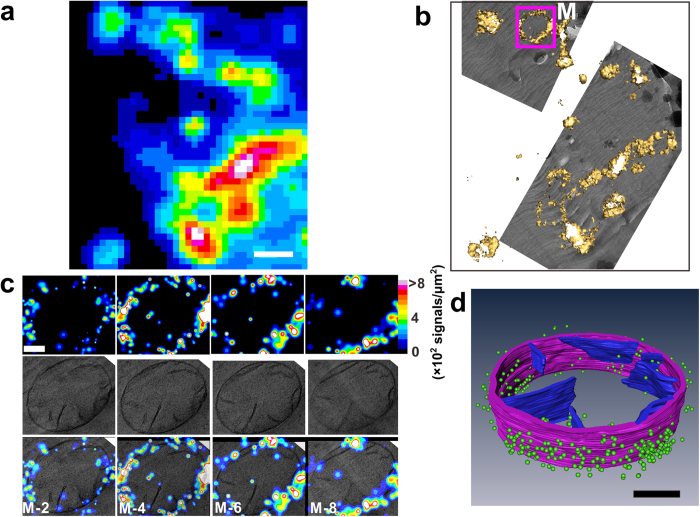
3D correlative images from cryo-sections of HEK293 cells expressing TOM20-Dronpa. (**a**) Summed image from single-molecule data. Scale bar, 1 μm. (**b**) Correlative isosurface reconstruction of a PALM image with low magnification TEM data. (**c**) Each column in c was from a single layer of 3D PALM (top), single layer of cryo-ET (middle) and correlative representations (bottom), respectively. The structural resolution of the whole 3D data set was estimated to be 73.7 ± 5 nm employing the Fourier ring correlation method^35^,^36^, corresponding to a density of 749 signals/μm^2^. Scale bar, 200 nm. (**d**) Correlative 3D segmentation of cryo-ET data with single-molecule localization of fluorescent proteins. Mitochondrial outer membrane and cristae are denoted by purple and blue, respectively. Dronpa molecules are denoted by green dots. For clear demonstration, we kept those signals that have higher localization precision (2.9/3.0 nm, mean/median), which rejects 90% of the signals. Scale bar, 200 nm.

Next, we correlated 3D fluorescence super-resolution imaging with 3D electron micrographs. We verified that the electron beam could bleach the FPs even at CT, so the correct order is fluorescence imaging first, followed by cryo-electron tomography (cryo-ET). After cryo-fluorescence super-resolution imaging, we transferred the specimens to a commercial cryo-TEM (Titan Krios 300 KV, FEI). We used the EM finder grids to locate the cells on which we had performed fluorescence imaging and collected EM data (Fig. 4a,b, **Methods**). Because formvar film is less electron conductive than carbon films, charge-induced movement and radiation damage of the electron beam may become issues of concern. We thus used low dose radiation and selected areas near the grid bar for tilted imaging to facilitate charge conducting. No clear charge-induced movement or radiation damage was observed under our experimental condition. The fine alignment between the fluorescent and electron images was achieved by three steps involving different fiducial markers, and the final registration error could be controlled at ~12 nm (Figure S8), and the comprehensive process was described in Methods section. As shown in Fig. 4c, a section of the mitochondrial outer membrane and cristae was clearly visualized by 3D reconstruction from cryo-ET (**Methods**, Movie S1), while super-resolution imaging of Tom20-Dronpa molecules showed correlated localization to the outer membrane at nanometer resolution (Fig. 4c,d).

## Discussion

Pioneering work has been conducted to correlate protein localization with ultrastructural features revealed by EM^4^,^5^,^19^,^22^. Compared to previous methods, our csCLEM method has the following advantages. 1) It avoids chemical fixation and contrast enhancement procedures that may cause artifacts in EM or damage the FPs^5^. 2) The cryo-condition not only preserves the specimen to near-native state but also enhances the photon budget due to reduced photobleaching. By using low fluorescence background formvar film instead of high absorbance carbon film, we were able to increase the power of the excitation laser and thus obtain ten times more photons (Table 1) than recently reported under cryogenic conditions^22^. The higher power of the activation laser also helps to identify a larger number of cryo-photoswitchable FPs. 3) Nanometer resolution is obtained in 3D for both cryo-SMLM and cryo-ET. Two identified red PA-FPs candidates, PATagRFP and PAmCherry, may also be combined with Dronpa or EGFP to achieve dual-color cryogenic superresolution. 4) The use of CEMOVIS enables the study of thick biological samples and offers very low out-of-focus background for super-resolution fluorescence imaging. In summary, we have extended CLEM to vitrified sections of mammalian cells at higher localization precision and within near-native cellular context. Our csCLEM method harnesses the super-resolution power of both SMLM and cryo-ET. The enhancement of axial resolution by introducing axial interferometry and the development of new dedicated fluorophores under CT could further extend the application of this approach. We envision that csCLEM will facilitate quantitative deciphering of functional protein organization and the relative spatial distribution to native cell architecture at unprecedented 3D resolution.

## Methods

### Protein expression and purification

The FPs were cloned into a pRSET vector using the appropriate restriction sites and expressed in the *Escherichia coli* strain BL21 (DE3). The bacteria were cultured at 310 K to OD = 0.6. The expression of proteins was induced by adding 0.1 mM IPTG and incubating overnight at 295 K. The proteins were purified with a Ni-NTA His-Bind resin (Qiagen). Then, further purification of the proteins was performed through a gel-filtration step using a Superdex 200 column (GE Healthcare). Purified proteins were concentrated by ultrafiltration and diluted in PBS for additional analysis.

### Spectra measurement

The measurement of spectra of Dronpa at different temperatures was performed using a spectrometer (FLS920, Edinburgh Instrument) equipped with a liquid nitrogen cryostat (OptistatDN-V2, Oxford Instruments). The slow-freezing of the samples from 293 K to 143 K was achieved by meticulous control of the liquid nitrogen flow. Purified Dronpa was diluted to ~0.02 mM. This concentration was necessary to avoid impurity peaks. Moreover, glycerol (60%) was used as a cryo-protectant to prevent crystalline ice formation. The evolution of the emission spectra of Dronpa subjected to a temperature gradient with excitation wavelength at 445 nm was observed (Fig. 2c,d).

### Plasmid construction

The Dronpa gene was cloned and inserted into the pEGFP-N1 (Clontech) vector using BamHI and NotI to replace the EGFP gene, which generated pDronpa-N1. The first 33 amino acids of TOM20 (Homo sapiens, NP_055580) were PCR-amplified and inserted into pDronpa-N1. The correct localization of TOM20-Dronpa in HEK293 cells was verified using confocal microscopy (**Methods**, Figure S5)

### Cell culture

HEK293 cells were cultured in Dulbecco’s Modified Eagle Medium (DMEM) (Gibco) containing 10% fetal bovine serum and maintained at 310 K and 5% CO_2_ in a humidified incubator. Cells were then transiently transfected using Lipofectamine^TM^ 2000 (Invitrogen) for 24 h.

### Cryo-nanoscopy setup

The cryo-nanoscopy system (Fig. 1a, **Methods**, Figure S1) consists of a cryo-chamber and a home-built upright microscopy (Figure S2a). We used a commercial cryo-chamber from Leica (Leica EM FC6) which is widely used for ultrathin cryo-sectioning of biological samples and has been shown to keep samples vitrified. The cryo-chamber uses cryogenic nitrogen gas to build up a stable cryogenic environment surrounding the sample stage and objective (Olympus , 100x, NA 0.8). Inside the cryo-chamber, a copper block (made of oxygen free copper) (**Methods**, Figure S2b), which is connected with the piezo stage by two glass rods, is used to support a copper sample holder (**Methods**, Figure S2c). The sample holder (made of oxygen free copper) has been designed to adopt four EM finder grids and is kept in place on the copper block by four small magnets integrated inside both parts. The glass rods is connected to a 3-axies piezo stage placed outside of the cryo-chamber. The piezo stage is attached to a XY stage. The goal of the design is to reduce the heat conduction from the cryo-chamber to sample stages and to provide high mechanical stability by avoiding the vibration of cryo-chamber due to liquid nitrogen pump. A heating wire is attached to a copper tube connecting the objective to prevent water condensation at the objective. For fluorescence activation and excitation, we used a 405 nm diode laser (OBIS, Coherent) and two solid-state lasers (OPSL, Coherent) with wavelengths of 488 nm and 561 nm coupled into a single-mode polarization-maintaining optical fiber. The laser beams were combined using DM1 and DM2 (LM01-427-25, LM01-503-25, SEMROCK) and delivered into the objective with DM3 (Di01-R405/488/561/635-25×36, SEMROCK), which reflected all laser beams but allowed the transmission of emitted fluorescence. An AOTF (AOTFnC-400.650, AA) was used for flexible control of the wavelength and intensity of the lasers delivered by the fiber.

Two EMCCDs (iXon DV-897 BV, Andor) were used in this implementation to ensure that the super-resolution imaging system (EMCCD1) and the feedback stabilization system (EMCCD2) remained independent of each other during data acquisition. A green (532 nm) light-emitting diode (LED) facilitated brightfield imaging. The fluorescence of the dark red beads (200 nm, Invitrogen) excited by the 488 nm laser was split by DM4 (DM690, Olympus), which reflected parts of the bead signal to EMCCD2 and allowed transmission of the leftover bead signal and the fluorescence of Dronpa. The image of the dark red beads and Dronpa on EMCCD1 was split by DM5 (FF560-Di01-25×36, SEMROCK) and cleaned by EM2 and EM3 (FF03-525/50-25, FF01-697/58-25), then projected onto two regions of EMCCD1. The bead image on EMCCD1 was used for correlation with TEM images, and the bead image on EMCCD2 was used for drift correction. The major advantage of the two-EMCCD system is the flexibility of optimizing the feedback stabilization system and fluorescence imaging system separately. Two cylindrical lenses were placed before EMCCD1 and EMCCD2 to enable imaging in 3D. A feedback loop that tracked the image of the dark red beads on EMCCD2 in real time actuated a piezo stage (P-733.3 DD, E-516 controller, Physik Instrument) and locked the sample at a fixed set point during data acquisition. Two computers were used to control EMCCD1 and EMCCD2 independently. A fast synchronization unit was designed to communicate with PC2 and the piezo stage controller to synchronize imaging with piezo stage movement for fast feedback. The fast feedback stabilizes the position of the sample with ~5 nm precision during data acquisition (Fig. 1b–e).

### Cryo-nanoscopy imaging and data analysis

EM finder grids with cryo-sections were first transferred to a sample holder that can support up to 4 grids in liquid nitrogen. Then, the sample was transferred into the cryo-chamber and mounted onto a copper block by four tiny magnetic buttons. A tiny Pt100 temperature sensor was inserted into the copper block around the samples to monitor the temperature. The cryo-chamber was usually pre-cooled to 113 K for more than 30 min before specimens were loaded. We first identified regions with fluorescence under low-power illumination with a 488 nm laser and further verified the regions of interest (ROIs) by the bright-field image on EMCCD1. Then, we ensured the presence of fiducial markers (fluorescent beads) on the red channel of EMCCD1 and EMCCD2 and locked the specimens at set positions through our feedback stabilization system. Next, we gradually increased the 488 nm laser power to 1.5 kW·cm^−2^ at the specimen to switch the ensemble Dronpa fluorescence to the single-molecule level. The 1.5 kW·cm^−2^ power was chosen as a compromise of imaging speed and avoiding devitrification of the specimen. Finally, the single-molecule images were obtained at 20 Hz. The activation laser pulses ranged from 0.005–0.5 kW·cm^−2^ at the specimen during data recording with a 4 Hz repeat rate and 0.01 s width. To avoid build-up of heat, we divided our imaging process into several sessions. Each session lasts for 5 min or less, with 10–20 seconds of breaks in between with no laser illumination. Inside the cryo-chamber, samples are surrounded by continuous replenished, gently flowing cryogenic nitrogen gas. The heat caused by laser-sample interaction would be quickly conducted during the breaks. At the end of the experiments, we have checked our sample and verified that there is no obvious crystalline ice formation. One example with 17 min total laser illumination is presented in Movie S1.

Data analysis and super-resolution image reconstruction were performed as previously described with some modifications^3^,^25^,^31^. We first applied an àtrous wavelet filter^32^ to the original fluorescence images to extract all the single molecules. Then, a trajectory linking algorithm (http://physics.georgetown.edu/matlab/) was used to find the corresponding single molecule in successive frames. Each identified molecule was background-subtracted and fit to an elliptical Gaussian function to obtain its precise centroid position and Gaussian width (w_x_ and w_y_). Based on the calibration curve (**Methods**, Figure S7), the molecules whose ellipticity was too large or too small were rejected. The remaining molecules were divided linearly into 8 z-sections on the basis of z-positions. Meanwhile, sample drift during image acquisition in all three dimensions was further estimated by analyzing the trace of fiducial markers which are imaged on red channel of EMCCD1. Finally, every drift-corrected molecule was represented by a Gaussian spot whose FWHM was determined by the localization error of each molecule, and the final PALM image was rendered.

To analyze the properties of FPs under CT, purified FPs were first diluted to ~1 mg/ml and fast frozen under a Vitrobot Mark IV (FEI). The excitation laser power was maintained at ~2.0 kW·cm^−2^. Single-molecule data were recorded at CT and analyzed as previously described^33^.

To analyze the behaviors of fluorescent proteins under CT, we collected fluorescence images under different illumination protocols (Fig. 2a,b, **Methods** and Figures S3, S4). We developed software for the control of AOTF and the generation of sophisticated time sequences of different lasers (https://github.com/shepherd87/pyAOTF).

### Preparation of support film

The traditional support film on an EM grid has a high background fluorescence that affects the performance of fluorescence nanoscopy. We found that most of the background fluorescence was generated by contaminants on the surface of the glass slides that were used to generate the thin formvar film. We applied a chemical cleaning method to wash the glass slides. Briefly, glass slides were washed in a mixture of 250 ml of clean water, 50 ml of ammonium hydroxide, and 50 ml of hydrogen peroxide in a 500 ml beaker for 3 h at 383 K. After rinsing 3 times in ddH_2_O, the glass slides were carefully transferred to a beaker containing anhydrous ethyl alcohol overnight and dried on a clean bench. A cleaned glass slide was dipped into a 0.2% formvar/chloroform solution in a volumetric beaker and dried above the liquid surface. After scoring the edge of the slides, the film was floated onto the water surface, and 200 mesh EM-finder copper grids (Gilder Grids, England) were placed on the film and dried on filter paper.

For fine correlation between light and EM, we deposited 200 nm fluorescent beads and 50 nm gold particles on the surface of the formvar film before applying to vitreous cryo-sections. Briefly, 3 μl of 1:300 diluted dark red 200 nm fluorescent beads was added to a glow-discharged grid for 1 min, and extra liquid was then blotted using filter paper. After the grid was dried, another 3 μl of a ddH_2_O-diluted solution of 50 nm gold particles (Microspheres-Nanospheres, 790116-010) was applied onto the grid for 1 min, and extra liquid was blotted using filter paper. The grids were then dried and stored until use.

### High-pressure freezing

Transfected HEK293 cells were harvested enzymatically by trypsin and centrifugation at 600 g, then re-suspended in an equal volume mixture of 40% w/v dextran (M_r_~40,000, Sigma) and 1:2 diluted dark red 200 nm fluorescent beads. Finally, the samples were vitrified using a high-pressure freezing apparatus (EMPACT2, Leica Microsystems, Germany) as described previously^18^.

### Cryo-electron microscopy of vitreous specimens

Cryo-EM of vitreous specimens has been thoroughly described^18^ and has been correlated with conventional fluorescence imaging^20^,^21^. In the current study, the vitrified sample was mounted in the holder of an ultramicrotome (UC6/FC6, Leica Microsystems, Germany) at 113 K. After trimming the sides of the copper tube with a cryo 45° diamond trimming knife (Diatome, Biel, Switzerland), the sample was sectioned using a cryo 25° diamond knife (Diatome, Biel, Switzerland) with a nominal thickness of 200 nm. Ribbons of sections were transferred onto the above-prepared grid using an eyelash glued to a wooden stick, and the sections were pressed and then attached to the formvar film using two polished ceramic plates. The grids with cryo-sections were stored in liquid nitrogen prior to observation.

After fluorescence imaging, TEM sample grids containing vitreous cryo-sections of HEK293 cells were transferred and imaged by a cryo-electron microscope (FEI Titan Krios) operated at 300 kV and equipped with a 4K*4K CCD camera (Gatan UltraScan 4000). For cryo-ET data collection, a defocus of approximately -15 μm was used with a total electron dose <7000 e/nm^2^.

Single-axis tilt series were collected covering the angular range of −60° ~ +60° in 2° increments with a nominal magnification of 18000×. Tilt series were aligned, and tomograms were generated using the Inspect3D software (FEI). During reconstruction, raw images were binned once, resulting in a final voxel size of 1.92 nm.

To confirm that the cryo-sections remained vitreous after fluorescence microscopy imaging, we performed an illumination-dependent devitrification assay verified by electron diffraction (**Methods**, Figure S6). For this purpose, the sections were loaded onto a Gatan 626 cryo-holder maintained at 93 K and then transferred to a TEM (FEI Talos F200C) operated at 200 kV and equipped with a field emission gun and a 4K*4K Ceta camera. TEM images were obtained under a low-dose condition at a dose rate of ~500 e/nm^2^. Then, diffraction images were recorded with a selective area aperture of 200 μm, camera length of 530 mm and exposure time of 1 s. We found that the sample remained mostly vitrified when the illumination intensity was up to 1.75 kW·cm^−2^ at the specimen. When the laser intensity was increased to 2.0 kW·cm^−2^, we began to observe devitrification, with clear diffraction spots from small ice crystals.

Employing formvar film instead of carbon film may lead to charging and beam-induced specimen movement. We used the following methods to minimize this problem. (1) We tried to image samples near the grid bar where cryo-sections attached better than at the center of the hole. Moreover, cryo-sections near the grid bar have better conductivity than the ones at the center of the hole. (2) Brightfield light microscopy images and fluorescence images helped us to locate the same regions with high precision, which significantly reduced the searching time under EM and thereby minimized the charging and radiation damage of the specimen. (3) Low dose radiation was adopted during EM data collection.

### Alignment of cryo-SMLM with EM

Currently, two methods have been introduced to correlate cryo-SMLM images with EM images. The first method utilizes fiducial markers, whereas the second method uses the image details from both light microscopy and EM.

The first method is usually applied for the alignment of 2D cryo-SMLM images with a single TEM projection image. We used electron dense fluorescent beads, which were visible under light microscopy (LM) and EM, as well as gold particles (**Methods**, Figure S8). The entire registration progress involves three steps: registration between two fluorescent channels, registration between dark red fluorescent signals and low-magnification electron micrographs, and registration between low-magnification and high-magnification micrographs. Each time before cooling the cryo-SMLM system, we deposited diluted four-color fluorescent beads onto a cover glass and used it for the registration between the single-molecule channel and the dark red channel. This type of registration error is denoted by ε_l_. During correlative imaging, we used electron dense fluorescent beads to map the dark red fluorescent channel to low-magnification EM micrographs. This type of registration error is denoted by ε_1−e_. Finally, we used fluorescent beads and gold particles to map low-magnification electron micrographs to high-magnification electron micrographs, and this type of registration error is denoted by ε_e._ The final registration error is calculated as follows:


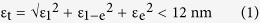


For the alignment of 3D cryo-SMLM images with electron tomography, we developed a simple but efficient registration method. Briefly, we first extracted the outer membrane of the mitochondrion of interest from EM images using segmented line tools in *ImageJ* (denoted as a closed curve). Then, we calculated the distance between each single molecule using the extracted outer membrane (denoted by ***D**i*, 1 ≤ ***i*** ≤ ***N***, where **N** is the total number of single molecules). We shifted (Δx, Δy) and rotated (θ) the extracted line to find the best parameters that minimize the sum of all distances (Σ*Di*). The description above can be submitted to the following optimization problem:


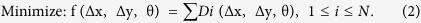


The molecules that were too far from the extracted outer membrane were excluded from calculation.

For the z-axis, we first calculated the histogram of the z-position of all the single molecules, which followed a Gaussian distribution. Then, we used the peak position and FWHM of the Gaussian distribution to indicate the middle layer and the thickness of the vitreous sections, respectively. The z-distribution of single molecules was 240 nm, which was comparable with the thickness calculated from the electron tomogram of 220 nm.

In practice, we randomly selected 2/3 of all detected single molecules to perform the registration procedure. Then, we calculated the distances between the remaining molecules and the extracted outer mitochondrial membrane. The histogram of these distances shows that the average distance between Dronpa molecules and the outer mitochondrial membrane is approximately 8.6 nm, which is comparable to the immune-gold staining results^34^.

## Additional Information

**How to cite this article**: Liu, B. *et al.* Three-dimensional super-resolution protein localization correlated with vitrified cellular context. *Sci. Rep.* **5**, 13017; doi: 10.1038/srep13017 (2015).

## Supplementary Material

Supplementary Information

Supplementary Movie S1

## Figures and Tables

**Table 1:** Single molecule statistics of selected FPs under cryogenic conditions.

Fluorescent Proteins	Total Photon Number		Localization Error (nm)	
	Mean	Median	Mean	Median
EGFP	895 ± 165	584 ± 108	14.7 ± 0.5	13.7 ± 0.6
mEos3.2 (green)	843 ± 227	312 ± 153	14.0 ± 1.1	13.0 ± 0.6
mEos3.2 (red)	1334 ± 502	614 ± 370	13.5 ± 4.6	12.1 ± 3.1
PATagRFP	621 ± 60	260 ± 18	17.8 ± 0.3	18.3 ± 0.1
PAmCherry	1026 ± 24	454 ± 18	15.0 ± 0.1	14.5 ± 0.1
Dronpa	2203 ± 316	1576 ± 227	10.1 ± 1.2	8.9 ± 1.4
mGeos-M	1814 ± 164	954 ± 35	10.6 ± 2.2	9.0 ± 1.7

Fluorescent protein samples were prepared as described in **Methods**.
