# Supplementary material for: Three-dimensional super-resolution protein localization correlated with vitrified cellular context
Source: Sci Rep. 2015 Oct 14;5:13017. doi: 10.1038/srep13017 (PMC4604464; doi:10.1038/srep13017)
Supplement: Supplementary Information [file srep13017-s1.pdf]

SUPPORTING INFORMATION

Three-dimensional super-resolution protein localization correlated with vitrified cellular context

Bei Liu[1,2,*], Yanhong Xue[1,*], Wei Zhao[1,3,*], Yan Chen[1,3], Chunyan Fan[1,3], Lusheng Gu[1,2], Yongdeng Zhang[1,2], Xiang Zhang[1], Lei Sun[1,4,], Xiaojun Huang[1,4], Wei Ding[1,4], Fei Sun[1,3,4], Wei Ji[1,3,4] and Tao Xu[1,2,3,4]

[1]*National Laboratory of Biomacromolecules, Institute of Biophysics, Chinese Academy of Sciences, Beijing, 100101, China.* [2]*College of Life Science and Technology, Huazhong University of Science and Technology, Wuhan, Hubei, 430074, China.* [3]*College of Life Sciences, University of the Chinese Academy of Sciences, Beijing, 100049, China.* [4]*Center for Biological Imaging, Institute of Biophysics, Chinese Academy of Sciences, Beijing, 100101, China.*

Correspondence: Wei Ji, E-mail: jiwei@moon.ibp.ac.cn ; Tao Xu, E-mail: xutao@ibp.ac.cn

*These three authors contributed equally to this work.

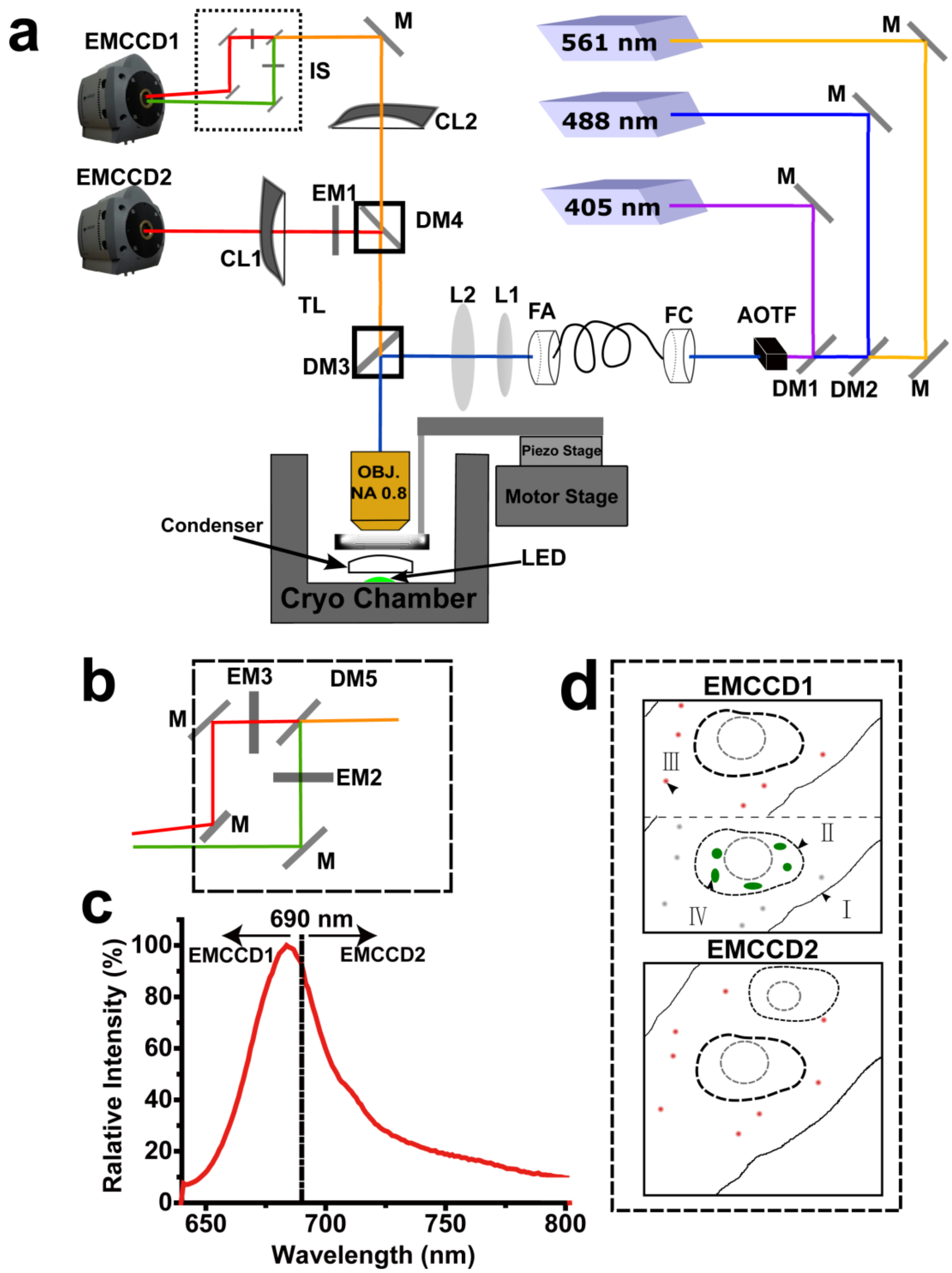

Figure S1. Schematic configuration of the cryo-nanoscopy system. Labels are defined here, and their functions are explained in **Methods**. M: mirror, DM: dichroic mirror, EM: emission filter, AOTF: acousto-optic tunable filter, FC: fiber coupler, FA: fiber adapter, L:

lens, CL: cylindrical lens, IS: image splitter, TL: tube lens. (**a**) Schematic representation of the cryo-nanoscopy setup. (**b**) Inside details of the image splitter. (**c**) Emission spectrum of dark red fluorescent beads. (**d**) Function declaration of the two EMCCDs illustrated by cartoon maps. The fluorescent signal collected by the objective was divided into two pathways by DM4, which were then projected to the two EMCCDs. **I** denotes the boundary of the vitreous section, **II** denotes the boundary of the cell, **III** denotes dark red fluorescent beads, and **IV** denotes the mitochondrion. The use of fluorescent beads that have overlapping emission spectra with the fluorescent protein, such as Dronpa, increases the alignment precision of dual-color images. A feedback loop that tracked the image of dark red beads on EMCCD2 in real time actuated a piezo stage and locked the sample at a fixed set point during data acquisition. This figure was drawn by Bei Liu.

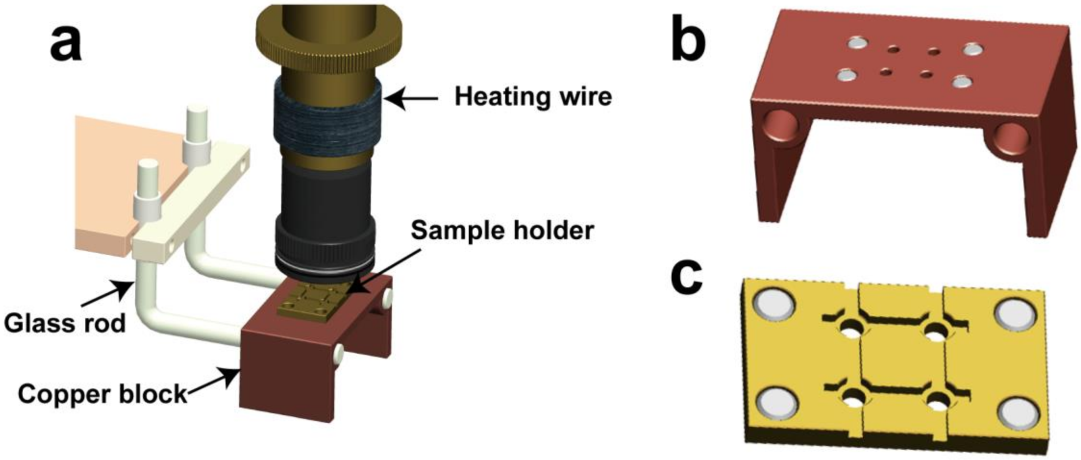

Figure S2. (**a**) Enlarged view of sample mounting and objective shown in Figure 1a. Magnified view of the copper block (**b**) and sample holder (**c**) in **a**. This figure was drawn by Wei Ji.

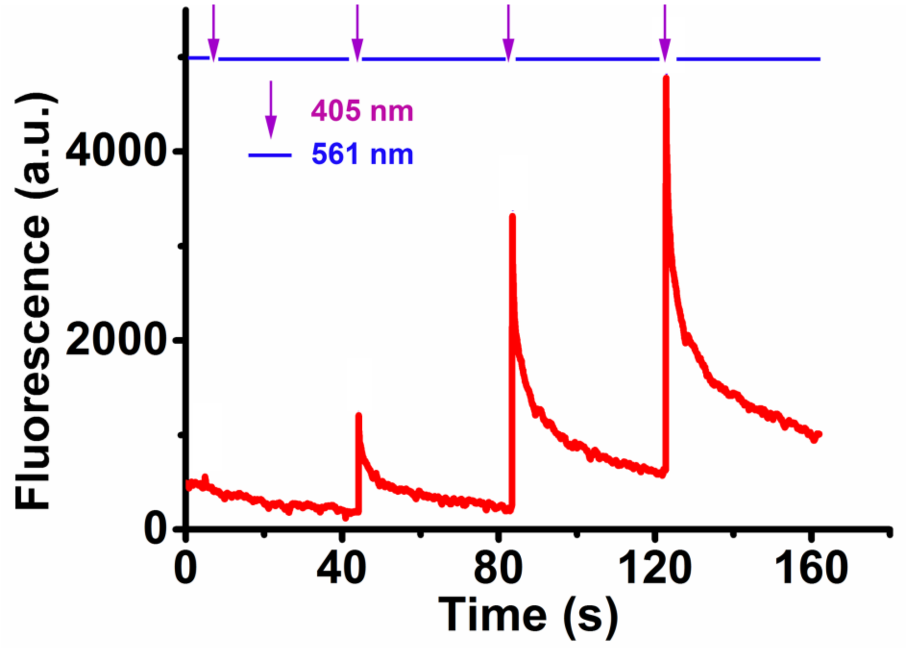

Figure S3. Photoconversion capability of mEos3.2 at CT. Purified mEos3.2 proteins can be converted to the red form by a strong 405 nm laser under cryogenic conditions with a set temperature of 113 K. Each purple arrow indicates a 5 s 405 nm laser pulse with the power increased gradually (0.1 mW, 1 mW, 5 mW, and 10 mW, respectively). The power of the 561 nm laser was maintained at 50 mW. The laser power was measured at the rear pupil of the objective. At RT, the laser power required for efficient photoconversion is usually less than 0.04 mW[1].

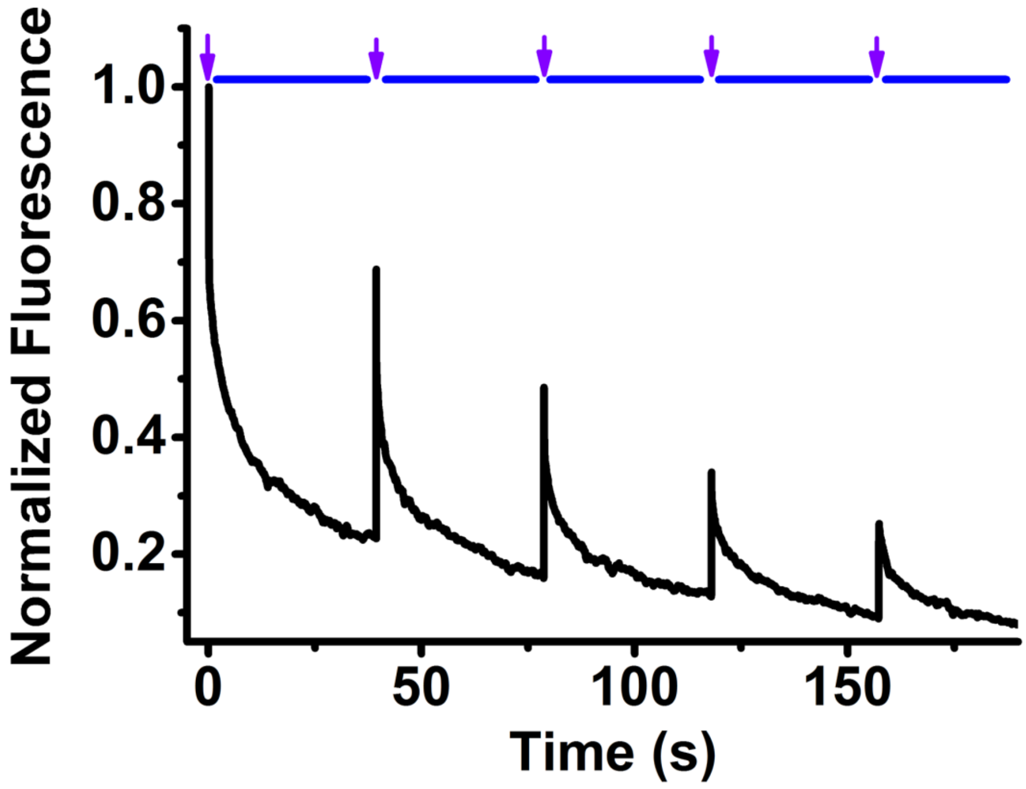

Figure S4. Photoswitching capability of EGFP at CT (113 K). Purified EGFP proteins were first switched off by a 488 nm laser, followed by alternative illumination with 405 nm and 488 nm lasers. Each purple arrow indicates a 5 s 405 nm laser pulse.

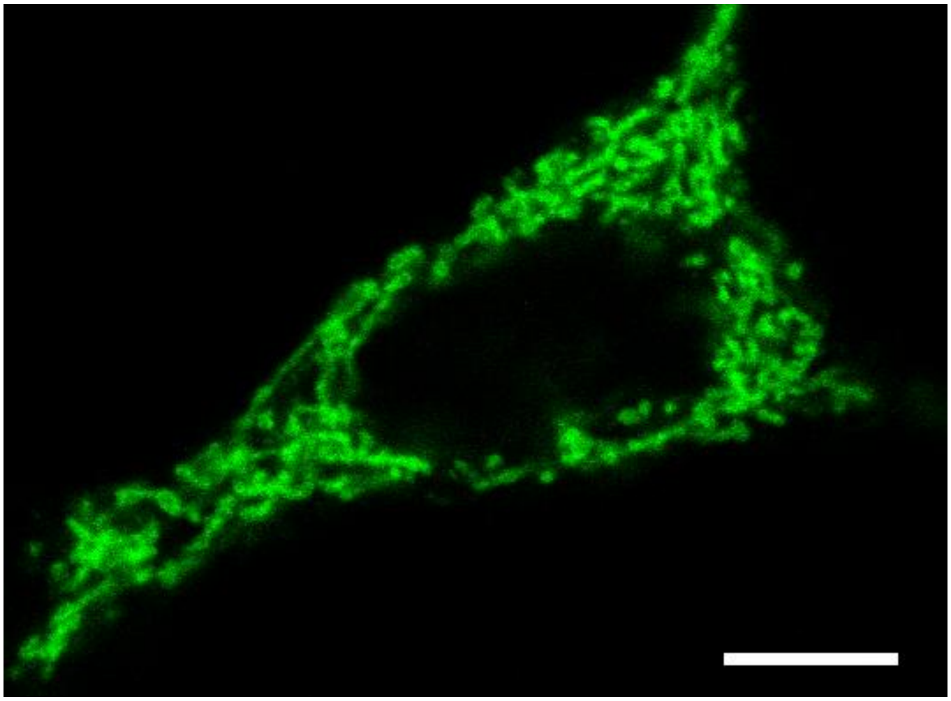

Figure S5. Localization validation of TOM20-Dronpa in a HEK293 cell by confocal microscopy. Scale bar, 10 μm.

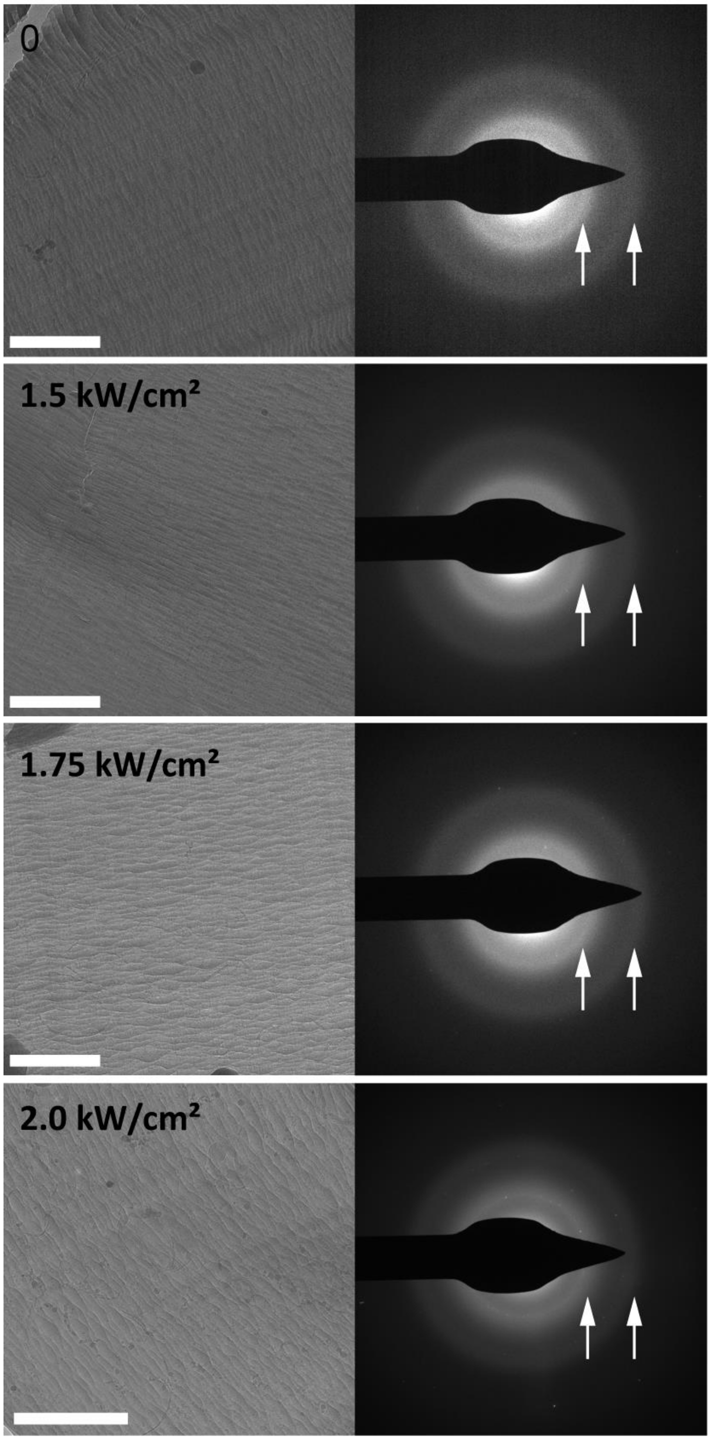

Figure S6. Electron diffraction of cryo-sections of a vitrified culture cell. Cryo-sections of vitrified culture cells (20% dextran added as a cryo-protectant) were illuminated by a 488 nm laser with varied power: 0, 1.5, 1.75, and 2.0 kW·cm^{-2}. After a 5 min illumination, the sections were loaded onto a Gatan 626 cryo-holder maintained at 93 K and then

transferred to a TEM (FEI Talos F200C) operated at 200 kV and equipped with a field emission gun and a 4K*4K Ceta camera. TEM images were obtained under low-dose conditions at a dose rate of ~500 e/nm^2 (left column, scale bar: 1 μm). Then, diffraction images were recorded with a selective area aperture of 200 μm, camera length of 530 mm and exposure time of 1 s. The two dominant diffuse rings (0.21 nm^{-1} and 0.36 nm^{-1}) representing the typical diffraction pattern of vitreous ice are indicated by arrows. Significant diffraction spots from crystallized ice can be observed for the sections illuminated with high laser power (2.0 kW·cm^{-2}) but are rarely found at lower illumination power, indicating that the laser illumination power of 1.5 kW·cm^{-2} is safe for avoiding devitrification.

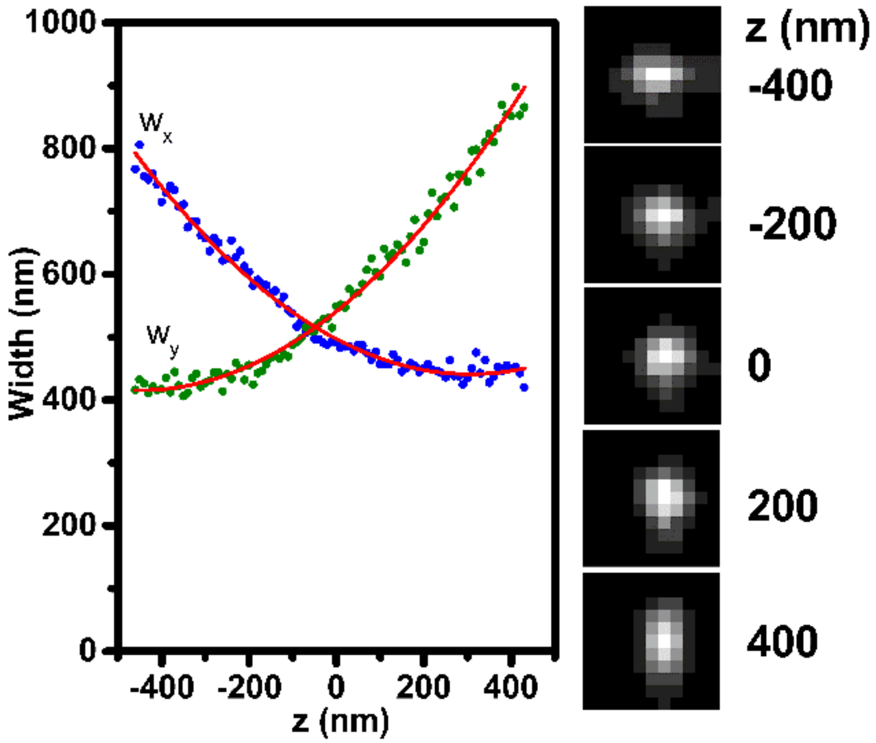

Figure S7. Calibration curve used for 3D super-resolution image reconstruction. Calibration curve of FWHM w_x and w_y obtained from 40 nm fluorescent beads. Right panel shows a series of images of a fluorescent bead at various z-positions.

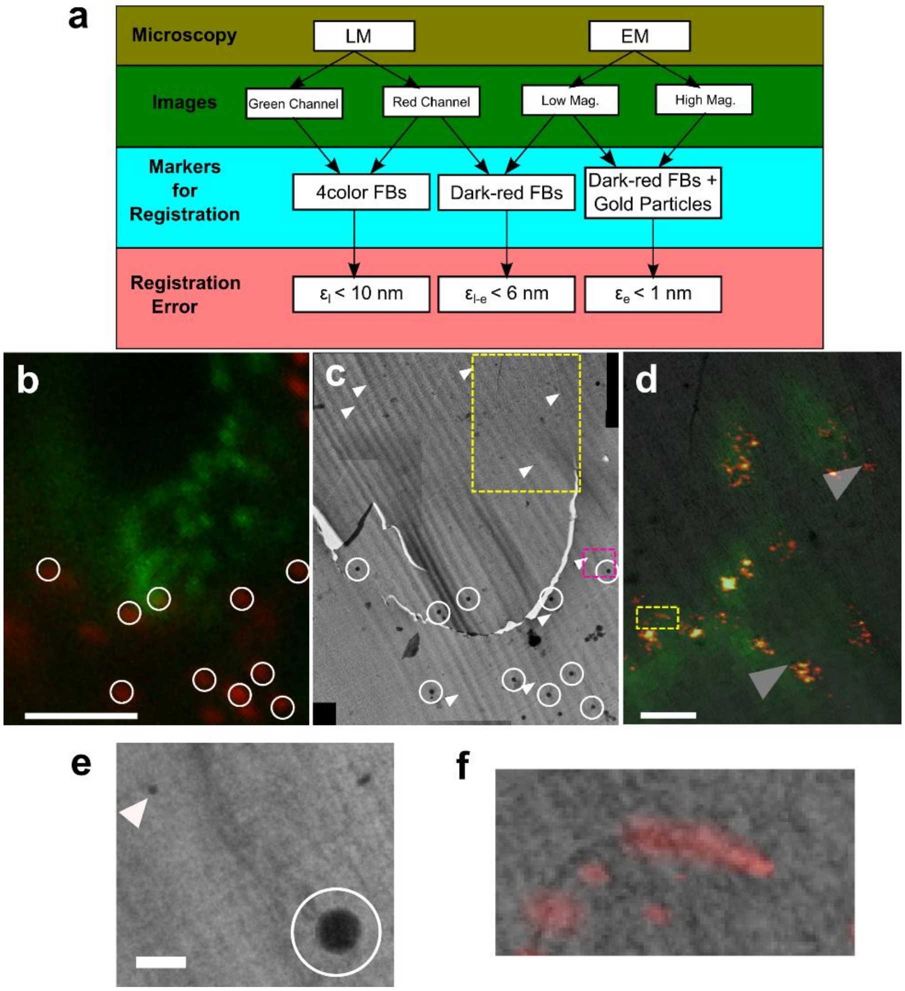

Figure S8 . Alignment procedures between cryo-SMLM and cryo-EM images based on fiducial markers. (**a**) Workflow of CLEM registration. Three types of fiducial markers were involved in the alignment process: 100 nm 4-color fluorescent beads (FBs), 200 nm dark red FBs, and 50 nm gold particles. Two fluorescent channels (Dronpa signals and dark red FBs) separated by optosplit were calibrated in advance using 4-color FBs on a clean formvar film. (**b**) Conventional fluorescence image of a vitreous section with overlapping green (Dronpa) and dark red (dark red FBs) channels according to the alignment results of 4-color FBs performed before imaging the vitrified samples. Scale bar, 5 μm. (**c**) Same area imaged by cryo-EM at low magnification. White circles indicate dark red FBs, which were used for registration between light microscopy and EM. White arrows indicate gold particles, which were used for

registration between the low- and high-magnification EM images. (**d**) Correlative cryo-SMLM and cryo-EM images of the yellow region in c. Scale bar, 1 μm. (**e**) Magnified image of the pink region in c. Scale bar: 200 nm. (**f**) Magnified image of the yellow region in d.

Movie S1: Tomographic slices of the cryo-section show in Figure 4.

REFERENCES

1 Zhang, M. *et al.* Rational design of true monomeric and bright photoactivatable fluorescent proteins. *Nat. Methods* **9**, 727-729 (2012).
